# Extended low-frequency phase of the distortion-product otoacoustic emission in human newborns

**DOI:** 10.1121/10.0003192

**Published:** 2021-01

**Authors:** Anders T. Christensen, Christopher A. Shera, Carolina Abdala

**Affiliations:** 1 Caruso Department of Otolaryngology, University of Southern California, Los Angeles, California 90033, USA; 2 Caruso Department of Otolaryngology and Department of Physics and Astronomy, University of Southern California, Los Angeles, California 90033, USA tcanders@protonmail.com, christopher.shera@gmail.com, Carolina.Abdala@med.usc.edu

## Abstract

At constant $f_{2}/f_{1}$ ratios, the phase of the nonlinear distortion component of the $2f_{1}-f_{2}$ distortion-product otoacoustic emission (DPOAE) has a steep low-frequency segment and a flat high-frequency segment in adults and newborns. In adults, recent work found that a third segment characterizes the phase at even lower frequencies. The present study tests whether the same is true of the newborn DPOAE phase. Newborn and adult phase curves are generally similar. However, as previously reported, phase-gradient delays at mid frequencies (the region of steepest phase slope) are 50% longer in newborns.

## 1. Introduction

Most of what we know about the mechanical basis for low-frequency hearing we know through direct probing in animal models of the auditory nerve and basilar membrane (Cooper and Rhode, 1995; Recio-Spinoso and Oghalai, 2017; Temchin *et al.*, 2008). Although technically daunting, these experiments suggest that the apical half of the cochlea might in some ways be considered functionally distinct from the basal half, almost as if the cochlea were comprised of two parts joined by a seam of a width corresponding to one seventh of the length of the chinchilla cochlea (Shera, 2001, 2007; Temchin *et al.*, 2012). Noninvasive measurements of otoacoustic emissions (OAEs) suggest that this idea holds true in humans as well (Shera and Guinan, 1999; Shera *et al.*, 2000). Emissions due to either of the two known mechanisms of generation—nonlinear distortion and linear reflection—abruptly change phase slope at frequencies associated with the middle of the cochlea (Abdala and Dhar, 2010, 2012; Moleti *et al.*, 2017; Shera *et al.*, 2010).

In a recent parametric study of both distortion- and reflection-source OAEs in human adults, we characterized the more salient features of this phase slope transition. Although earlier work characterized this phase pattern in the DPOAE phase-frequency function as having only one break (near 2.5 kHz if plotted as a function of *f*_2_; and near 1.5 kHz if plotted as a function of the DP frequency), our most recent work suggests it may be better characterized by two frequency breaks and three segments (Christensen *et al.*, 2020). The two breaks in the phase occur at mean *f*_2_ frequencies of 0.86 and 2.57 kHz in adults. Translated into human cochlear location via the Greenwood map (Greenwood, 1990), these frequencies delimit a region in the middle, covering about a fifth of its length.

Considering phase a function of log frequency, and the distortion emission a function of its generation site, *f*_2_, unifies the frequency of the breaks observed for distortion- and reflection-type emissions. Furthermore, the phase of distortion-type OAEs can be scaled by a specific $f_{2}/f_{1}$ ratio-dependent factor that effectively removes its dependence on the primary-tone ratio in the transition region. We have presented a model that captures these major features by introducing a reflecting region in the middle of the cochlea that only low-frequency waves would encounter [see Christensen *et al.* (2020)].

Immaturities in both reflection and nonlinear distortion OAE amplitude and phase have been observed in newborns; however, most of these can be accounted for (at least partially) by considering the effects of outer- and middle-ear immaturities. An exception to this is the immaturity of the phase-gradient delay in the apical half of the newborn cochlea, which cannot be so easily explained by conductive immaturities (Abdala and Keefe, 2012). At frequencies below the phase break, the phase gradient is significantly steeper in newborns, which amount to delays of almost a millisecond. The current work was initiated to further investigate cochlear immaturities in newborns and to test whether the infant phase-frequency function can be well-characterized by a three-segment, two-break model.

## 2. Methods

In each newborn, we implemented one of two different experimental DPOAE paradigms. The first paradigm was designed to measure over an extended low-frequency range, 1–2 octaves lower than has ever been measured in this population. This we refer to as the *low-frequency paradigm*. The second paradigm was designed to study the dependence of the phase on the ratio of the primary-tone frequencies. These ratios determine the overlap of the traveling waves evoked by the two primary tones, *f*_1_ and *f*_2_. We refer to this as the *ratio paradigm*. In adults we are able to do both, record at many ratios and extend the low-frequency range, but in newborns the noise is much higher and our time with each subject was limited. For this reason, we reduced the parameter space, including only three $f_{2}/f_{1}$ ratios, and assigned newborns to one or the other paradigm.

### 2.1 Subjects

A total of 20 term-born neonates were successfully tested. Thirteen newborns were tested using the low-frequency paradigm; 5 of these 13 had data that were acceptable for further analysis according to the SNR criterion. Seven newborns were tested using the ratio paradigm. Two of them had acceptable data across both frequency and ratios according to a requirement that 25% of the data be of a signal-to-noise ratio (SNR) higher than 6 dB. The main reasons an infant test failed included restlessness and excessive mobility, which produced elevated noise floors and unstable probe fittings. Due to the safety concerns related to the Covid-19 pandemic, the Infant Auditory Research Laboratory was closed to data collection mid-way through this study and we were unable to accrue additional neonatal data.

All newborn subjects had passed the auditory brainstem response hearing screening prior to participation and were tested with our DPOAE research protocol within 24–48 h of birth, with the exception of two newborns delivered by caesarean section, who were tested outside of this window but within the first month of life. As term-born infants, their gestational ages ranged from 37–40 weeks, and their mean post-conceptional age at test was 40.3 weeks. Their average birthweight was 3232 g; and their average 1- and 5-min Apgar scores (which range from 1 [worst] to 10 [best] and reflect neonatal health at birth) were 8.6 and 9, respectively. Participation was voluntary and consented by one parent prior to testing. All procedures were approved by the Internal Review Board of the University of Southern California.

During successful testing, the newborns were sleeping in an isolette with the ear probe sealed into the ear canal, its cable secured with surgical tape to both the isolette and a rolled-up diaper or swaddling blanket next to their head. With each newborn, we had up to one hour of testing time available; typically, 20–30 min of the session involved relatively peaceful sleep for testing.

### 2.2 Instrumentation and calibration

The measurements were carried out using the ER10X probe system (Etymotic Research Inc., Elk Grove Village, IL, USA). The system was connected to a laptop running Windows 7 and the ASIO driver via an RME Babyface Pro audio interface (RME, Haimhausen, Germany), the input-output delay of which was measured and compensated for. In-house developed software written in Matlab (Mathworks, Natick, MA, US) controlled the experimental protocol.

Stimuli were calibrated across frequency using the forward-pressure level (FPL) procedure described by Scheperle *et al.* (2008) and the phase was referred back to the eardrum using an estimate of the delay between the face of the ear probe and the eardrum. The ear canal OAE was calibrated using the emitted-pressure level (EPL) procedure described by Charaziak and Shera (2017). Stimulus levels were constant at $\left\{L_{1},L_{2}\}=\{65,55\right\}$ dB FPL. Recalibration was initiated whenever the ongoing estimates of those levels had shifted more than 2 dB.

### 2.3 Low-frequency paradigm

The low-frequency paradigm assessed the $2f_{1}-f_{2}$ distortion-product OAE over a wide range of *f*_2_ frequencies from 0.25 to 8 kHz. By comparison, we are not aware of any previous reports of DPOAE phase below $f_{2}=0.8$ kHz in newborns. For each subject, the primary frequency ratio $f_{2}/f_{1}$ was selected at random from the set {1.16, 1.22, 1.28}. The stimuli were upward frequency sweeps whose sweep rate doubled smoothly for every octave swept. Approximately half^1^ of the duration was spent in the lowest-frequency octave through 0.5 kHz, half of the remaining duration in the octave through 1 kHz, and so on. Averaging in bands a fraction of an octave wide then reduces the noise at low frequencies more than it does at high frequencies [see further details in Christensen *et al.* (2019)]. To further lower the noise at low frequencies, the upper boundary of the frequency range changed with increasing repetitions. For the first four repetitions, sweeps went all the way to 8 kHz, then for the next four only to 4 kHz, then for the next eight up to 2 kHz, and so on. In effect, as data collection progressed, much more of the total time was dedicated to the lowest octave where the noise was highest.

When a brief noise artifact was detected (a spike greater than 2 standard deviations beyond the median) the measuring algorithm immediately ramped off, rewound the frequency by backtracking ≃2 s before ramping back up to make a replacement measurement. No more than four consecutive attempts to replace an artifact were allowed. The experimenter could also pause the measurement at any point and initiate a recalibration if necessary. Since the noise was always quite high at low frequencies despite these efforts, we did not fix the number of repetitions or a target noise floor in advance. Instead, the algorithm simply continued collecting data until our time with the newborn subject was up.

### 2.4 Ratio paradigm

Whereas the low-frequency paradigm prioritized DPOAE measurements at low frequencies, the ratio paradigm targeted its dependence on the primary frequency ratio. Specifically, each subject had their DPOAEs measured at *all* three ratios—1.16, 1.22, and 1.28—although in a reduced frequency range of *f*_2_ from 0.5 to 8 kHz. The stimuli were logarithmic frequency sweeps at a rate of 0.5 oct/s. The sweeps were split into two concurrent segments so that the range from 0.5 to 2 kHz was presented simultaneously with that from 2 to 8 kHz. Near 2 kHz, the concurrent sweeps overlapped by 1/6 oct.

In this paradigm, the sweeps were also short enough that phase-rotation averaging could be implemented. Under this scheme, the stimulus presentation is comprised of three pairs of primary sweeps 1/3 cycle out of phase with each other, so that when averaged the primary tones cancel out but leave the distortion emission (Whitehead *et al.*, 1996).

After each stimulus presentation, the measuring algorithm picked the next ratio to present based on which of the three had completed the fewest successful repetitions so far. A repetition was considered successful if the noise was within 2 standard deviations of the median noise floor. This method of dynamically selecting the next ratio condition to be presented allowed us to run the measurement until the hour was up or the baby woke up, and still has a reasonable chance of collecting data at all ratios.

### 2.5 Data analysis

The raw data from both DPOAE paradigms were averaged over the repetitions and analyzed using the least squares fit algorithm that is well-known by now in the literature [see Long *et al.* (2008) and Abdala *et al.* (2015) for its application to DPOAEs]. The distortion component of the total measured DPOAE response at $2f_{1}-f_{2}$ was extracted by analyzing it in frequency bands 1/3-oct wide using recursive exponential windows overlapping by 87.5%. To isolate the nonlinear-distortion part of the total DPOAE, we took the inverse Fourier transform of the frequency response and zeroed any signal delayed in this transform by more than 3 cycles using another recursive exponential window. The noise was calculated as the root mean square level of the responses 5% above and below the $f_{\text{dp}}$ frequency of the distortion product.

From the resulting complex-valued frequency domain data, the distortion phase, $\phi_{\text{dp}}$, was obtained by unwrapping the angle and referencing it to the estimated phase of the stimulus sweeps. The phase-gradient delay was computed as $\tau (f_{\text{dp}})=-d\phi_{\text{dp}}/df_{\text{dp}}$; however, since the data are regarded as a function of *f*_2_, not of the $f_{\text{dp}}$ frequency where they were measured, we define a scaled version of the phase, $\hat{\phi_{\text{dp}}}=r/(2-r)\phi_{\text{dp}}$ (where $r=f_{2}/f_{1}$), which preserves the phase-gradient delay, $\hat{\phi_{\text{dp}}}(f_{2})=-d\hat{\phi_{\text{dp}}}/df_{2}=-d\phi_{\text{dp}}/df_{\text{dp}}=\phi_{\text{dp}}(f_{\text{dp}})$. In adults, and as we will see in newborns, this scaling reduces the dependence of the phase on the primary frequency ratio.

In the final step of the analysis we estimated the characteristic parameters of the distortion-OAE phase—break frequencies and phase-gradient delays. The method was described in detail by Christensen *et al.* (2020). In brief, a model curve consisting of three segments (straight lines on a log frequency axis) was fit to the data. After obtaining an initial estimate of the best parameters by computing the squared error between the model and the data for breaks at all frequencies in the data, the final parameters were obtained by minimizing that same error using fitnlm, a nonlinear optimization routine in Matlab. The *f*_2_ frequencies where the model lines intersected were taken as the low- and high-frequency break frequencies, $f_{\text{LF}}$ and $f_{\text{HF}}$, respectively. The negative slopes of the low-, mid-, and high-frequency line segments (denoted $N_{\text{LF}},\;N_{\text{MF}}$, and $N_{\text{HF}}$, respectively) gave estimates of the phase-gradient delays (here in periods of *f*_2_ because of the log frequency axis) across the corresponding range of frequencies.

As a metric for the quality of the fit we used the “reduced $\chi^{2}$,” denoted $\chi_{R}^{2}$, which is the standard mean squared error normalized to include a penalty based on the number of free parameters in the model (Christensen *et al.*, 2020; Taylor, 1997). We also report the standard *R*^2^ coefficient indicating how much of the variance is explained by the models.

## 3. Results

**Fig. 1.**
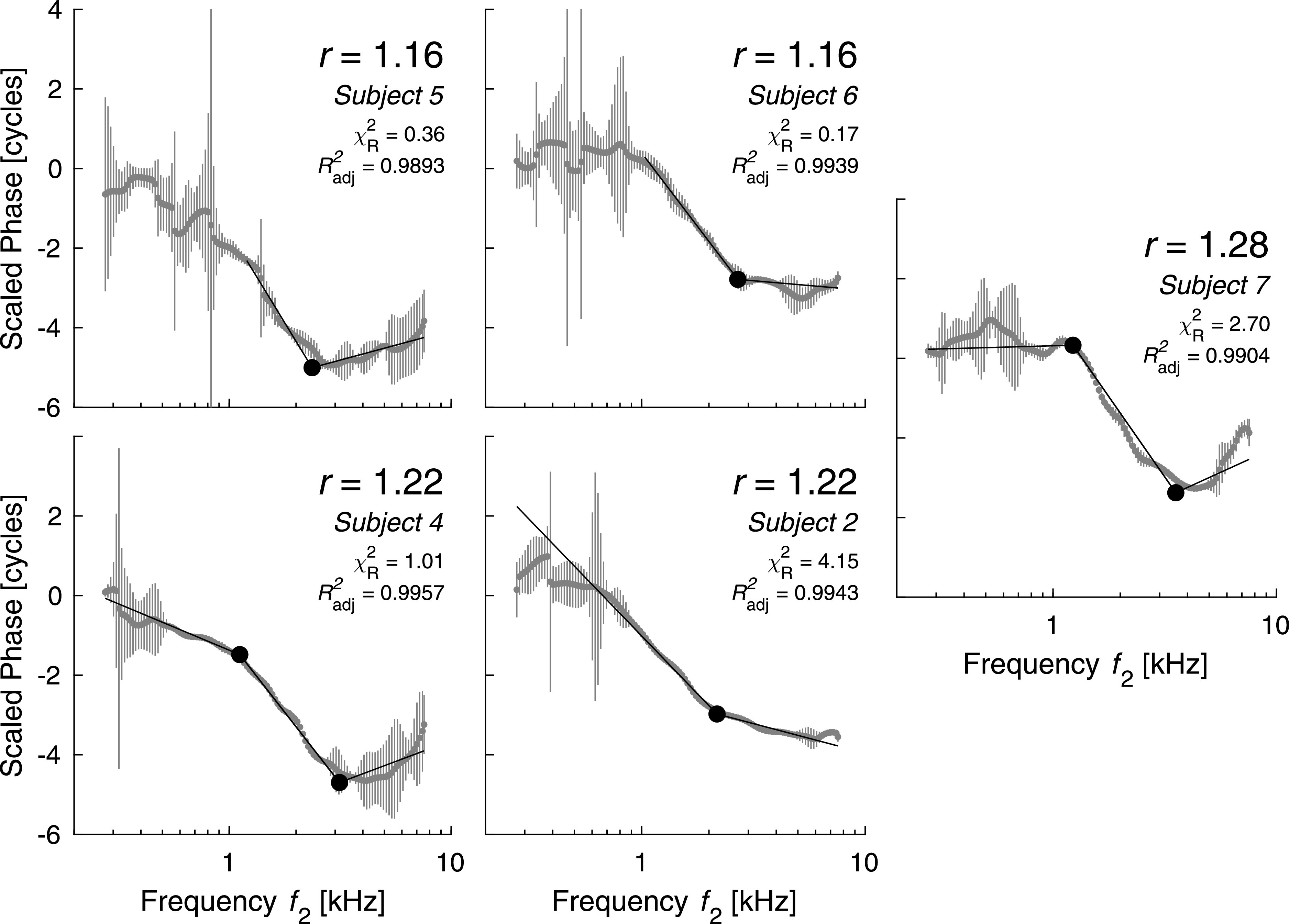
Straight-line models fitted to the scaled-phase data from the low-frequency paradigm. Only line segments covering a range of frequencies where the average SNR is higher than 6 dB are shown. Missing segments occur only at low frequencies, where the noise is highest and the uncertainty of the phase curve is unacceptable for meaningful interpretation. Also shown are the $\chi_{R}^{2}$ values for the quality of the fits, which are better the closer they are to 1, and the *R*^2^ coefficient indicating how much of the variance is explained by the models.

Figure 1 shows the phase results for the low-frequency paradigm with standard errors of the mean derived from the SNR. At *f*_2_ frequencies between 1 and 3 kHz the distortion-component phase is steepest, while at higher frequencies it is relatively flat, bending upward at the highest ratio. Superimposed on the data are the straight-line models we successfully fit to estimate the slopes and break frequencies of the phase in recent adult work (Christensen *et al.*, 2020). In adults the values of $\chi_{R}^{2}$ are usually within a factor of 2 of 1 (1 indicating the best possible fit), but in newborns they are only within a factor of 10. Nevertheless, the neonatal fits appear relatively good at *f*_2_ frequencies above 1 kHz. Subjects 4 and 7 have the best low-frequency DPOAEs, and both of these illustrate a striking similarity between neonatal and adult data: the emission phase of newborns “bends over” near 1 kHz, indicating a second break in addition to the one that has been consistently described near 2.5—3 kHz.

**Fig. 2.**
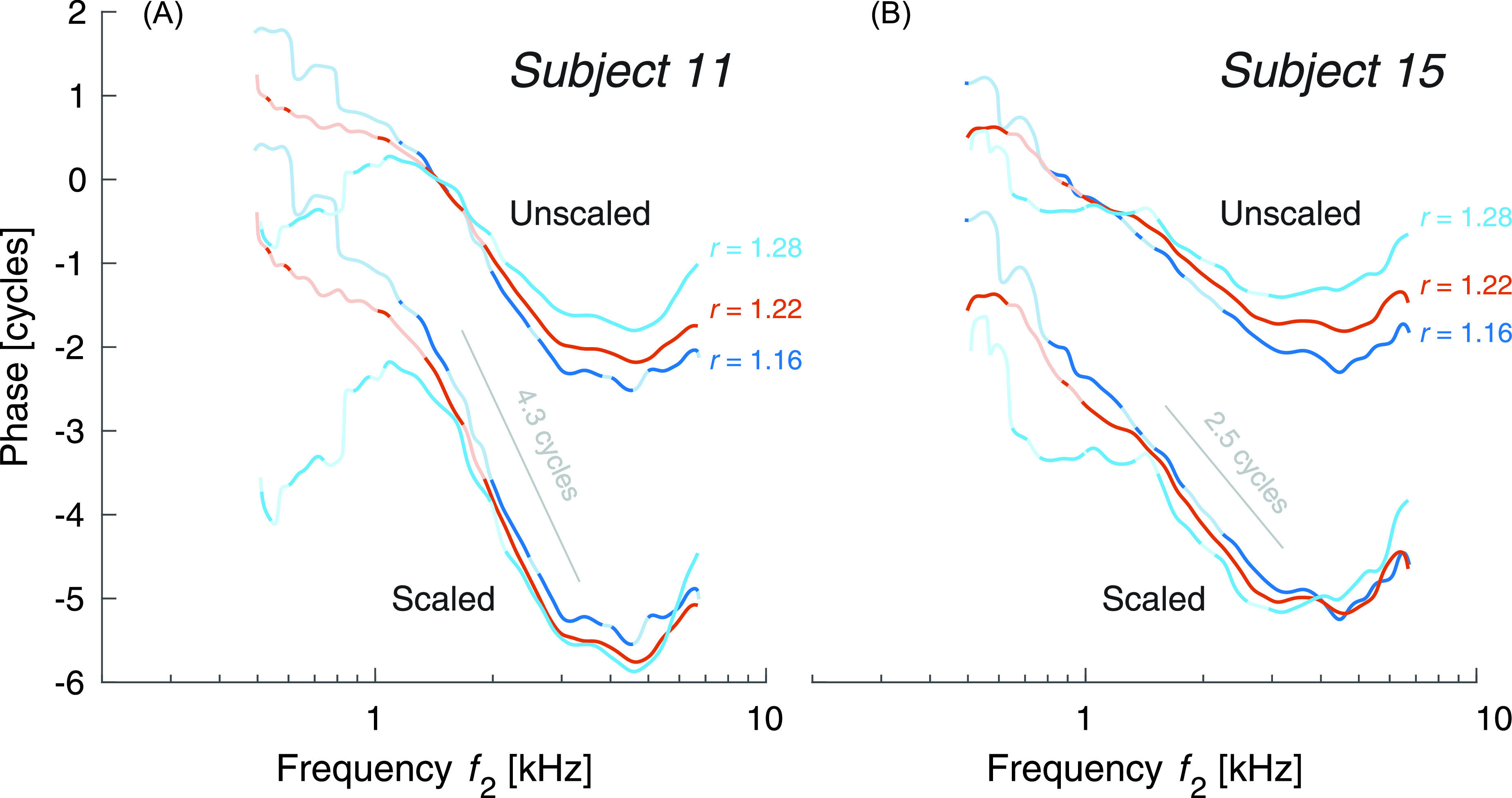
Distortion emission phase in two newborns at three ratios measured using the ratio paradigm. The frequency regions where the curves take on lighter colors have SNR less than 6 dB, meaning that the phase estimate may be unreliable. The phases are shown in both their original and scaled form. For the scaled phase, we multiplied the phase by the factor, r/(2−r). As described in the text, this largely removes the ratio dependence from the phase.

Figure 2 shows measurements in two newborns using the ratio paradigm. Varying the ratio alters the traveling-wave overlap associated with the generation of the DPOAE. Although noisy at low frequencies, the newborn data show the same ratio trends reported previously in adults (Christensen *et al.*, 2020); that is, when the phase is scaled by the ratio factor, r/(2−r), the mid- and high-frequency segments of the phase functions collapse onto each other, suggesting that the phase curves have little dependence on the ratio aside from this ratio factor.

That the phase increases with frequency at high frequencies is a reminder of the fact that phase-gradient delay is not simply the physical emission delay. Although phase-gradient delay is normally reported to be near zero, in adults we find that to only be the case at a ratio of 1.22 (Christensen *et al.*, 2020); at higher ratios it tends to be negative, while at narrower ratios it tends to be positive. Here, newborn phase gradients are positive at all three ratios, suggesting that the near-zero condition lies at a narrower ratio than in adults. The condition is thought to be brought about by the wave-fixed nature of the distortion source in combination with the scaling symmetric way in which the basilar membrane distributes waves along its length.

**Fig. 3.**
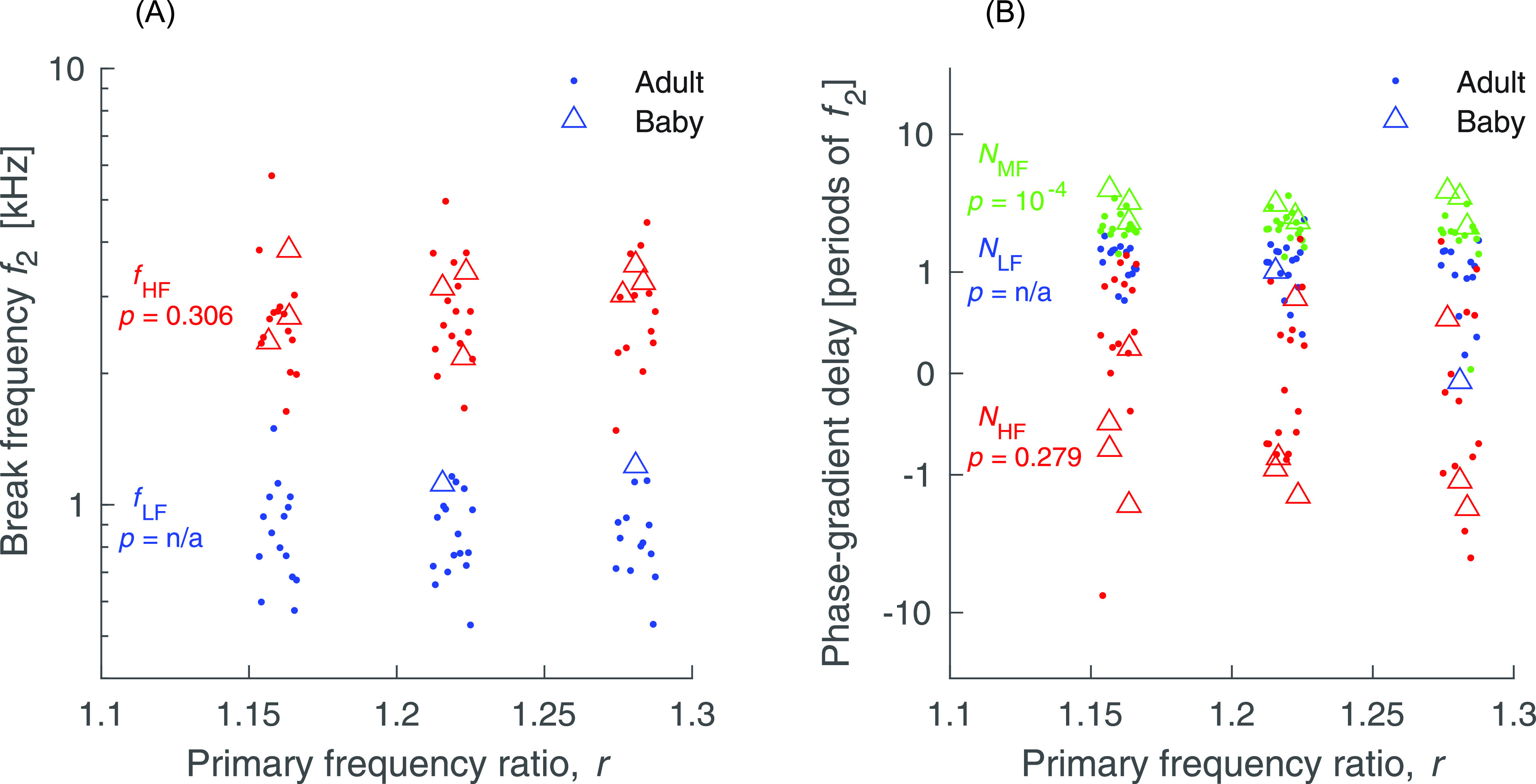
Comparison of characteristic parameters of distortion emission phase in human adults and newborns. Break frequencies shown on the left and phase-gradient delays on the right. The *p*-values shown are the result of one-way analyses of variance comparing adults and newborns. Between the two groups, only the mid-frequency delay, $N_{\text{MF}}$, is significantly different. Although not shown, our analyses also suggest that none of the baby parameters depend significantly on the primary frequency ratio.

Figure 3 summarizes how the *f*_2_ break frequencies and phase-gradient delays in newborns compare to those in adults. The newborn delay and break frequency data are also tabulated in Table 1. The parameters that are estimated best are the high-frequency break frequency, $f_{\text{HF}}$, and the mid- and high-frequency delays, $N_{\text{MF}}$ and $N_{\text{HF}}$, respectively. Break $f_{\text{HF}}$ does not depend on the ratio (*p = *0.11; one-sample *t*-test). Therefore, as in adults, the data may be collapsed across the ratio. As shown well in Fig. 3, the break frequency $f_{\text{HF}}$ in newborns is not different from that in adults (*p = *0.31; one-way ANOVA); the high-frequency delay, $N_{\text{HF}}$, is also not different in newborns and adults (*p = *0.28). The mid-frequency delay, $N_{\text{MF}}$, however, is significantly longer in newborns ($p=2\times 10^{-4}$). In newborns, this delay is 2.99 periods of *f*_2_, while in adults it is 2.03 periods across the three ratios.

**Table 1.** Summary of estimated break frequencies and emission delays in all 7 newborns tested successfully. Low-, mid-, and high-frequency segments with SNRs lower than 6 dB were not good enough for parameter estimation, and so, these are marked “n/a” in the table. Frequencies, $f_{\text{LF}}$ and $f_{\text{HF}}$, are given in kHz while the units of the phase-gradient delays, $N_{\text{LF}}$, $N_{\text{MF}}$, and $N_{\text{HF}}$, are dimensionless (in periods of *f*_2_).

	*r*	$\chi_{R}^{2}$	*R* ^2^	$f_{\text{LF}}$ [kHz]	$f_{\text{HF}}$ [kHz]	$N_{\text{LF}}\;[\cdot ]$	$N_{\text{MF}}\;[\cdot ]$	$N_{\text{HF}}\;[\cdot ]$
Subject 5	1.16	0.365	0.9893	n/a	2.36	n/a	3.97	–0.65
Subject 6	1.16	0.174	0.9939	n/a	2.69	n/a	3.21	0.21
Subject 2	1.22	4.147	0.9943	n/a	2.17	n/a	2.53	0.64
Subject 4	1.22	1.011	0.9957	1.12	3.14	1.01	3.11	–0.91
Subject 7	1.28	2.696	0.9904	1.23	3.54	–0.07	3.50	–1.11
	1.16	0.705	0.9931	n/a	n/a	n/a	n/a	–0.41
Subject 11	1.22	13.799	0.9722	n/a	n/a	n/a	n/a	–0.75
	1.28	4.466	0.9590	n/a	3.03	n/a	3.86	0.45
	1.16	1.797	0.9300	n/a	3.83	n/a	2.30	–1.67
Subject 15	1.22	13.619	0.9160	n/a	3.41	n/a	2.32	–1.43
	1.28	0.732	0.9242	n/a	3.23	n/a	2.12	–1.75

Despite substantial differences in methodology, the overall results of the present study are consistent with the phase data reported by Abdala and Dhar (2010, 2012). These investigators were first to quantify parameters of the two-segment phase-frequency function and the frequency of the conjectured break in adults and newborns. In particular, they found that the break frequency was similar between adults and newborns, while the newborn low-frequency phase-gradient delay (our mid-frequency delay) was significantly longer, on the order of 1 ms. To convert the roughly 1-period difference found in the present study into milliseconds, we can divide it by the edge frequencies of the frequency region it represents, $f_{\text{LF}}$ to $f_{\text{HF}}$. This gives a phase-gradient delay in the range from 1.16 to 0.39 ms (assuming that $f_{\text{LF}}$ is the same in newborns as it is in adults). Abdala and Dhar suggested that this difference might indicate an immaturity in the apical half of the newborn cochlea.

## 4. Discussion and Conclusions

This work has further defined DPOAE phase characteristics in neonates. Although limited in quantity, the data included are of good quality and met our SNR criteria. Additionally, the results are consistent with two documented newborn findings: (1) newborns have steeper DPOAE phase gradients (i.e., longer delays) in the apical half of the cochlea and (2) phase break frequencies are generally similar throughout the human lifespan (Abdala and Dhar, 2012). These consistent findings act to validate the newborn DPOAE data collected here. The prolonged delay for low-mid-frequency DPOAE data cannot be easily attributed to middle-ear immaturities. Outer- or middle-ear immaturities in newborns are expected to attenuate stimulus transmission through a relatively inefficient conductive system [see Abdala and Keefe (2012)]. Therefore, due to the lack of level-dependence shown by DPOAE phase, middle-ear immaturities cannot explain the non-adult-like steep phase gradient (Abdala *et al.*, 2011). The longer delays in the neonatal cochlea are consistent with a recent temporal bone study of the newborn cochlea, reporting morphological immaturities in the infant organ of Corti, such as a thicker and wider basilar membrane, which could produce prolonged delays (Meenderink *et al.*, 2019).

More targeted to the research question in the current study, the DPOAE phase-frequency functions from the two newborns with good SNR at the lowest frequencies are in agreement with similar data from adults. In particular, the trend in these functions is well characterized by a three-segment line with two break frequencies, rather than one, as has previously been suggested. The two $f_{\text{LF}}$ break values that could be reliably estimated in the present study were somewhat higher than found in adults. Whether this apparent difference bears out when more data are obtained remains to be seen. The other new contribution of the present study was measurements of the phase-frequency functions in newborns at ratios other than 1.22, namely 1.16 and 1.28. These ratio measurements exhibit the same trends as those found in adults. Furthermore, as in adults, scaling the phase by the factor r/(2−r) substantially reduces the ratio dependence of the phase. Our recent phenomenological model (Christensen *et al.*, 2020), which explains this feature well, may also help account for the distortion-OAE phase breaks seen in newborns.

## Footnotes

^1^ To be more precise: 51.61%.
